# The Use of Exenatide in Managing Markers of Cardiovascular Risk in Patients with Type 2 Diabetes: A Systematic Review

**DOI:** 10.3390/ijerph13100941

**Published:** 2016-09-23

**Authors:** Omorogieva Ojo

**Affiliations:** Faculty of Education and Health, University of Greenwich, Avery Hill Campus, London SE9 2UG, UK; o.ojo@greenwich.ac.uk; Tel.: +44-20-8331-8626; Fax: +44-20-8331-8060

**Keywords:** exenatide, type 2 diabetes, cardiovascular risk, glycated haemoglobin, glycaemic control, metformin

## Abstract

*Objective*: This review examines the use of exenatide twice daily in managing changes in markers of cardiovascular risk in patients with type 2 diabetes. *Background*: Type 2 diabetes is a progressive metabolic disorder, which results from defects in insulin secretion and/or insulin action leading to chronic hyperglycaemia and associated cardiovascular complications. Despite the use of diet, exercise, oral antihyperglycaemic agents and insulin, the progressive nature of the condition means that the levels of the preventive and treatment measures would have to be increased and/or new therapies have to be developed in order to address the long term impact of type 2 diabetes. The advent of exenatide, a glucagon-like peptide-1 receptor agonist provides a useful basis for managing type 2 diabetes and related cardiovascular complications without the side effects of regular diabetes therapies. However, exenatide twice daily is often used in combination with other therapies, although the mechanism of exenatide in managing diabetes and and associated cardiovascular risks and complications remain complex and still evolving. *Method*: A range of databases including EBSCOhost online research database were used to access articles based on PICO (Population, Interventions, Comparative Interventions, Outcomes) framework and Boolean operators. Results: Eleven randomised controlled studies which met the inclusion criteria were selected for this review. Nine of the eleven studies showed significant decrease in body weight among participants in the exenatide group compared with placebo or control group while the other two studies did not report statistically significant differences in body weight. In adition, all the studies showed statistically significant decrease in glycated haemoglobin (HbA1c) in the exenatide group compared to controls except in one study. In the present review, the seven studies, which looked at the effect of exenatide twice daily on lipid profile, did not find any significant difference between the exenatide group and the control group except for High density lipoprotein-cholesterol in two of the studies. However, statistically significant decrease was observed between exenatide group and controls with respect to blood pressure (systolic and/or diastolic) in these studies. *Discussion*: It would appear that exenatide is more effective in reducing body weight in patients with type 2 diabetes when used in combination with metformin than when used alone or in combination with thiazolidinedione. The findings of this review would suggest that exenatide twice daily may be useful in managing cardiovascular risks and complications by reducing body weight, HbA1c and blood pressure.

## 1. Introduction

Type 2 diabetes is a progressive metabolic disorder of multiple aetiology resulting from defects in insulin secretion from the beta cells and/or insulin resistance in peripheral tissues leading to chronic hyperglycaemia [1].

Patients with type 2 diabetes are at greater risk of developing cardiovascular disease (CVD). According to Saraiva and Sposito [2], about 68% of people over 65 years of age with diabetes die of some form of cardiovascualr disease and CVD deaths amongst adults with diabetes are two to four times higher than those without diabetes. Although some observational studies have demonstrated that a higher glycated haemoglobin (HbA1c) level was associated with increased risks of cardiovascular diseases and deaths, the underlying mechanisms remain complex [3,4].

The progressive nature of type 2 diabetes despite the use of diet, physical activity and current therapies, such as metformin, sulfonylurea and insulin, may explain why therapeutic requirements tend to increase with time [1,5]. Therefore, other strategies have to be developed in order to control the resulting hyperglycaemia and associated complications although researchers continue to debate which combination therapies will be effective in controlling hyperglycamia [5]. For example, Jansson et al. [6] demonstrated the protective effect of low-cost diabetes drugs on the negative influences of hyperglycaemia regarding all cardiovascular diseases combined and myocardial infarction. The addition of sulfonylurea and thiazolidinedione as treatment for diabetes are useful alternatives, however, these may have their limitations including the problems of hypoglycaemia, their tendency to elicit weight gain and oedema with potential to reduce patient compliance [1,5]. Therefore, new therapies, which do not have the usual side effects of the current therapies, have to be developed.

The National Institute for Health and Care Excellence (NICE) guideline [7] in the UK clearly outlined the algorithm for blood glucose lowering therapy in adults with type 2 diabetes. According to the guideline, if a triple therapy (such as metformin, a Dipeptidyl peptidase-4-inhibitor and sulfonylurea) is not effective, not tolerated or contraindicated, then, it will be useful to consider combination therapy with metformin, a sulfonylurea and a Glucagon-like peptide-1 (GLP-1) mimetic for adults with type 2 diabetes. To qualify for this treatment, the patients should have body mass index (BMI) of ≥ 35 kg/m^2^ or have a lower BMI than 35 kg/m^2^ and for whom insulin therapy would have significant occupational implications, or weight loss would benefit other significant obesity related co-morbidities. Similar approach in the form of algorithm for the initiation and adjustment of therapy in the management of hyperglycaemia in patients with type 2 diabetes has been developed [8]. This framework is based on consensus between the American Diabetes Association and the European Association for the Study of Diabetes [8]. 

The advent of the use of GLP-1 based therapies has brought new dimension in the management of type 2 diabetes and associated cardiovascular complications. According to McCormack [9], human GLP-1 is produced by L-cells of the intestinal mucosa and is an incretin, a gastrointestinal hormone responsible for the enhanced secretion of insulin from the beta cells of the pancreas in response to food intake. In addition, GLP-1 receptor agonists, such as exenatide, significantly decrease glycated haemoglobin through suppressing glucagon production, delaying gastric emptying, and reducing appetite and food intake by increasing satiety [10].

Exenatide is a 39-amino acid peptide and is an incretin mimetic, which was the first of the new incretin mimetic class of antihyperglycaemic agents to be marketed in the US and European Union [9,11]. Exenatide is a short acting agent, which can be administered subcutaneously twice daily (exenatide BID) although the extended release formulation can be administered once weekly (exenatid QW) [9].

There is evidence that exenatide has some advantages over current therapies for patients with type 2 diabetes. It does decrease the risk of severe hypoglycaemia when used in the absence of insulin or sulfonylurea and it is associated with reductions in body weight in the absence of diet and exercise programme [11,12]. It has been reported that GLP-1 can stimulate the proliferation and neogenesis of beta cells and may also inhibit their apoptosis [13]. In addition, GLP-1 decreases gastrointestinal motility, which could extend the entry of nutrients to be absorbed by the gastrointestinal tract and thus controls postprandial glucose levels and ensure normal glucose homeostatis [2]. Impairment in the secretion of GLP-1 has been considered in the pathophysiology of type 2 diabetes and the administration of GLP-1 receptor agonists, which restores GLP-1 function has been shown to promote insulin secretion, sensitivity and enhance long term prognosis including reduction in cardiovascular risks and complications [14].

In patients with type 2 diabetes, the incretin effect is significantly impaired due to reduced production of GLP-1, metablolism and/or impairment of their actions [13]. The incretin effect has been defined as the difference in insulin secretion from an oral gluscose load compared with intravenous glucose administration and results from the observation that intrajejunal glucose enhances greater insulin release than intravenous glucose administration [2,15].

In view of the above, this is a systematic review of randomised controlled studies aimed at evaluating the potential role of exenatide and combination treatments on physiological and biochemical markers of cardiovascular risk in patients with type 2 diabetes. The objective of this review is to examine the use of exenatide twice daily in managing changes in markers of cardiovascular risk in patients with type 2 diabetes.

## 2. Methods

This review involved a literature search including a general scoping of the data bases which found previous reviews relevant to the population and intervention of interest, but these were either more than 5 years old, were narrative reviews or included other GLP-1 agonists, such as liraglutide, and/or included studies which were not randomised. The current systematic review is based only on randomised controlled studies. 

The literature search strategy for this review relied on previously published guidelines for reviews [16,17] and was based on the PICO framework; P (Population—patients with type 2 diabetes), I (Interventions—exenatide twice daily), C (Comparative interventions—control group, placebo) and O (Outcomes—Glycaemic control, glycated haemoglobin, microvascular complications, macrovascular complications, cardiovascular complications). A number of databases including EBSCOhost online research database, encompassing Academic search premier, Medline, Psychology and Behavioural sciences collection, PsycINFO®, SPORTDiscuss and Cumulative Index to Nursing and Allied Health Literature (CINAHL) Plus were accessed.

In addition, “Boolean” operators (and/or) allowing the combination of search terms and alternative search terms were used; “exenatide“ and “diabetes“ and “cardiovascular diseases“; “exenatide“ and “diabetes“ and “glycaemic control“; “GLP-1“ and “diabetes“; “GLP-1“ and “diabetes“ and “cardiovascular diseases“; “GLP-1“ and “diabetes“ and “macrovascular diseases“; “exenatide“ and “cardiovascular risks“; “exenatide“ and “cardiovascular complications“; “exenatide“ and “glycaemic control“; “exenatide“ and “diabetes“ or “glycaemic control“; “exenatide“ and “diabetes“ or “GLP-1“. References of articles were also reviewed to search for relevant studies. 

### 2.1. Inclusion Criteria

Studies included were those written in English and published between 2010 and 2016 only. In addition, only randomised controlled studies were selected for review.

### 2.2. Exclusion Criteria

Studies which did not meet the inclusion criteria outlined above were excluded from the review. The original hits were filtered and narrowed down using the above criteria.

### 2.3. Quality Assurance

The evaluation process involved assessing the quality of the research articles and this was based on the Scottish Intercollegiate Guidelines Network (SIGN) checklist for critical appraisal [18] and expert review of the articles.

### 2.4. Data Analysis

On the basis of the inclusion and exclusion criteria, 19,065 articles including full articles and abstracts were initially found following a search of the databases. However, based on the use of various search terms, this was significantly narrowed to smaller numbers. Of these, 11 articles which met the requirements for selection were included in the review (Table 1).

## 3. Results

All the eleven studies [19,20,21,22,23,24,25,26,27,28,29] (Table 1) for this review were multicentre and randomised controlled studies involving patients with type 2 diabetes. While seven of the studies [19,21,22,23,25,27,28] were conducted in at least 2 countries, the remaining 4 [20,24,26,29] were conducted in Italy, Japan, the US and Germany respectively. However, all eleven studies had background treatment in addition to the intervention treatments except the study by Gastaldelli et al. [19]. The background treatments included metformin and/or insulin glargine, pioglitazone, diet and exercise, and thiazolidinedione. Other background treatments were sulfonylurea and/or biguanide, thiazolidinedione and/or metformin. The background treatments were treatments taken by participants before the commencement of the study and/or during study (Table 1).

The sample size of the studies ranged from 54 to 1019 while the length of study ranged from 12 weeks to 4½ years. The duration of diabetes of the patients in the studies ranged from 1 ± 2 to 12 ± 7 years (Mean ± SD). 

### 3.1. Exenatide Twice Daily versus Placebo 

Exenatide twice daily was compared to placebo in seven of the 11 studies [19,20,21,22,23,24,25] (Table 1).

Participants in the exenatide groups with metformin as one of the background treatments showed statistically significant decrease in body weight in five of the studies [20,21,22,23,24] compared to placebo group. However, mean reductions in body weight were not statistically significant between exenatide and placebo groups in the studies by Gastaldelli et al. [19] and Liutkus et al. [25].

In addition, the exenatide group in all the seven studies showed statistically significant decrease in HbA1c compared with the placebo group except for one study [23], where HbA1c reduction was not significantly different between the two groups (*p* = 0.26). 

There were no significant differences in lipid profile between the exenatide groups and the placebo group [20,22,24,25] except with respect to HDL cholesterol in the study by Kadowaki et al. [24]. That study showed that significant reduction in HDL cholesterol was statistically greater in both exenatide groups compared to placebo although no statistically significant difference was observed in total cholesterol, LDL-cholesterol and triglycerides. However, systolic and diastolic BP decreased significantly in the exenatide group compared to placebo group in two studies [20,22]. In another study [25], diastolic pressure was significantly reduced in both treatment groups, while systolic blood pressure remained relatively unchanged from baseline values.

### 3.2. Exenatide Twice Daily versus Other Controls

While one of the remaining 4 studies had placebo + lifestyle modification as control [26], the other 3 studies were not placebo controlled [27,28,29]. The interventions included; exenatide + lifestyle modification versus placebo + lifestyle modification with metformin and/or sulfonylurea as background treatment [25]; exenatide versus Glimepiride with metformin as background treatment [27,28]; exenatide versus premixd insulin aspart with metformin as background treatment [29].

In all the 4 studies [26,27,28,29] (Table 1), there were significantly better clinical outcomes in the exenatide groups compared with glimepiride, premixed insulin aspart, placebo plus lifestyle modification programme, based on the specific outcomes measured by each study (body weight, blood pressure, lipid profile and glycaemic control). For example, there was significant decrease in body weight in exenatide group compared to control groups in all the four studies.

With respect to HbA1c targets (<7% and <6.5%) exenatide twice daily was non inferior compared to premixed insulin aspart [29]. In addition, there were significant differences in favour of the exenatide groups compared with the glimepiride group with respect to HbA1c < 7% (*p* < 0.0001) and HbA1c < 6.5% (*p* = 0.0001) [28]. The percentage of hypoglycaemia in the exenatide group was significantly (*p* < 0.0001) lower than the glimepiride group [27,28]. When compared with the placebo plus lifestyle, the exenatide plus lifestyle groups had significantly more participants who had HbA1c ≤ 6.5% (*p* = 0.001) [26].

The study by Simo et al. [27] showed that a statistically significant proportion of exenatide treated patients achieved the HDL-cholesterol goal than glimepiride treated patients at 36 months (*p* = 0.021). In addition, systolic blood pressure (BP) [26,27,28] and diastolic BP [26,27] decreased significantly in the exenatide groups compared with controls.

## 4. Discussion

The exact mechanisms of the potential benefit of exenatide in the control of cardiovascular risks and complications are complex and still evolving [12,13]. However, the findings of this review would suggest that the influence of exenatide in possibly managing cardiovascular risks and complications may be through its effect in reducing glycated haemoglobin (HbA1c), reducing body weight and blood pressure. These are the markers of cardiovascular risk [27]. This view is supported by previous reports, which revealed that the probable effects of exenatide may be through glycaemic control based on the link between HbA1c and cardiovascular events and evidence that tight glycaemic control and/or decrease in HbA1c has been shown to reduce microvascular risks and complications [1,9,12].

High levels of glycated haemoglobin have been proposed as independent risk factors for patients with diabetes and those without diabetes [30]. In addition, higher HbA1c levels are known to be positively correlated with future risk of stroke and coronary heart disease, and that for every 1% rise in HbA1c, there is an associated 8% increased risk of first cardiovascular event [3,31]. In the present review, all the studies showed statistically significant decrease in HbA1c in the exenatide group compared to controls except in the study by Gill et al. [23]. Perhaps the sample size with only 54 participants in the Gill et al. [23] study was not large enough to elicit any statistically significant difference in the HbA1c level compared to the other studies with sample sizes ranging from 79 to 1019 participants. The Simo et al. [27] study had no report on HbA1c levels.

Another possible mechanism of the likely benefit of exenatide on cardiovascular risks and complications is its impact on body weight. Based on the results of the studies reviewed, it is clear that nine of the eleven studies showed significant decrease in body weight among participants in the exenatide group compared with placebo or control group. Only two studies [19,25] did not report statistically significant differences in body weight between exenatide group and control group. While one of these studies had no background treatment [19], the other had thiazolidinedione, metformin plus thiazolidinedione [25]. Therefore, it would appear that exenatide is more effective in reducing body weight in patients with type 2 diabetes when used in combination with metformin than when used alone or in combination with thiazolidinedione. Increases in body weight and body mass index in patients with type 2 diabetes have been reported to increase the risk of cardiovascualr diseases whereas reduction in body weight increases insulin sensitivity and reduces blood pressure [12,32]. In addition, exenatide twice daily is superior to insulin, sulfonylureas and rosiglitazone in decreasing body weight [9].

Another mechanism of the potential action of exenatide on cardiovascular risks and complications may be its effect on the pathogenic link between type 2 diabetes and atherosclerosis [13]. The formation of advanced glycation endproducts (AGE) in patients with type 2 diabetes plays a key role in vascular damage and exenatide has been reported to ameliorate this deleterious effects of AGEs [13].

According to Anagnostis et al. [13], the main mechanism of the antihypertensive role of exenatide may be due to its effect on weight loss although the authors also alluded to the vasodilatory effects of GLP-1. Although exenatide twice daily may improve blood pressure and certain lipid parameters, changes are often small and variable between studies [9,33]. In the present review, the studies, which looked at the effect of exenatide twice daily on lipid profile [20,22,24,25,26,27,28], did not find any significant difference between the exenatide group and the control group except for HDL-cholesterol [24,27]. However, statistically significant decrease was observed between exenatide group and controls with respect to blood pressure (systolic and/or diastolic) in these studies. 

With respect to glycaemic control, episodes of hypoglycaemia were less frequent in the exenatide group compared to glimepiride [27,28] and premixed basal insulin aspart [29] groups. This appears to be in tandem with previous reports which suggest that the incidence of hypoglycaemia is relatively low with exenatide treatment except when used in combination with a sulfonylurea due to their mechanism of action, which is glucose dependent [15].

## 5. Conclusions

Based on the findings of this review, it would appear that exenatide twice daily may be useful in managing cardiovascular risks and complications by reducing body weight, HbA1c and blood pressure. In particular, the combination of exenatide with metformin was more effective in reducing body weight than using exenatide alone.

## Figures and Tables

**Table 1:** Summary of studies.

Citation	Country	Length of Study	Study Type	Sample Size	Age (Years)	Diabetes Duration (Years)	Background Treatment	Intervention	Glycaemic Control	Lipid Profile	Blood Pressure and Heart Rate	Outcomes/Body Weight
Gastaldelli et al. [19]	4 countries (Multi-centre)	24 weeks	Randomised Controlled Study	79	Exenatide (10 μg) 59 ± 2	2 ± 3 (exenatide 5 μg)	–	Exenatide twice daily versus placebo	Compared to placebo, 24 weeks of daily high or low dose exenatide treatment significantly reduced HbA1c (*p* < 0.01) and fasting plasma glucose (*p* < 0.05)	–	–	There were no differences in weight changes between the two exenatide and placebo groups
					Exenatide (5 μg) 57 ± 2	2 ± 3 (exenatide 10 μg)						
					Placebo 54 ± 2	1 ± 2 (placebo)						
Derosa et al. [20]	Italy (Multi-centre)	4 years	Randomised Controlled Study	174	57.1 ± 7.6	7.8 ± 3.2	Metformin (given for 8 months before randomly assigning patients), diet and exercise advise (commenced at baseline)	Exenatide twice daily versus placebo	Exenatide + metformin were better than placebo + metformin in decreasing HbA1c at 12 months (*p* < 0.05). Similar trend was recorded for fasting blood glucose.	No variation in lipd profile were observed in either of the 2 groups	Systolic and diastolic BP were not changed by placebo + metformin, but decreased by treatment with exenatide + metformin at 12 months compared with point of randomisation (*p* < 0.05)	Body mass and BMI obtained after 9 months and 12 months of exenatide + metformin were lower than the ones obtained for placebo + metformin group (*p* < 0.05 and *p* < 0.01 respectively)
Rosenstock et al. [21]	5 countries (Multi-centre)	30 weeks	Randomised Controlled Study	259	59 ± 9 (exenatide)	12 ± 7 (Exenatide)	Insulin glargine, metformin, pioglitazone Treatments given to participants for at least 3 months before study commenced and continued throughout study period.	Exenatide twice daily versus placebo	Exenatide participants had greater HbA1C reductions compared with placebo participants at end point (*p* < 0.001)	–	–	Exenatide participants lost more weight (*p* < 0.05)
					59 ± 10 (placebo)	12 ± 7 (placebo)						
Buse et al. [22]	5 countries (59 centres)	30 weeks	Randomised Controlled Study m	259	59 ± 9 (exenatide)	12 ± 7 (exenatide)	Insulin glargine with or without metformin or pioglitazone (or both agents). Treatments given to participants for at least 3 months before study commenced and continued throughout study period.	Exenatide twice daily versus placebo	HbA1c level decreased by 1.74% with exenatide and 1.04% with placebo (*p* < 0.001). Proportion of participants who had minor hypoglycaemia were similar in both groups	Concentration of triglycerides, LDL, HDL did not differ between the groups	Systolic and diastolic pressures decreased (*p* < 0.01 and *p* < 0.001) respectively from basline with exenatide. Heart rate increased from baseline in the exenatide group	Weight decreased by 1.8 kg with exenatide and increased by 1.0 kg with placebo
					59 ± 10 (placebo)	12 ± 7 (placebo)						
Gill et al. [23]	Canada and Netherlands (Multi-centre)	12 weeks	Randomised Controlled Study	54	Exenatide 57 ± 11	7 ± 4 (exenatide)	Metformin (given for 30 days and continued during study), thiazolidinedione (given for 120 days and continued during study), metformin + thiazolidinedione (continued during study).	Exenatide twice daily versus placebo	HbA1c reduction was not significantly different between the 2 groups (*p* = 0.26)	–	Differences in heart rate between the 2 groups was not significant (*p* = 0.16). Exenatide therapy showed trends towards lower systolic BP, but similar diastolic BP between the groups.	There were significant differences (*p* < 0.05) in body weight between exenatide and placebo group.
					Placebo 54 ± 10	6 ± 4 (placebo)						
Kadowaki et al. [24]	Japan (23 centres)	24 weeks	Randomised Controlled Study	179	58 ± 10	12.2 ± 6.3 (exenatide 5 μg)	Sulfonylurea, Sulfonylurea and biguanide, sulfonylurea and thiazolidine derivative. Treatments given to participants for 90 days before screening.	Exenatide twice daily versus placebo	The changes in HbA1c levels were significantly greater (*p* < 0.001) in both exenatide groups than the placebo group.	The reduction in HDL cholesterol was statistically greater in both exenatide groups (5 μg, *p* = 0.020 and 10 μg *p* = 0.014) than placebo group. No statistically significant differences were observed in total cholesterol, LDL cholesterol and triglycerides	–	Reduction in body weight were significantly greater (*p* = 0.026) in exenatide 10 μg group, than placebo group.
						11.6 ± 7.0 (exenatide 10 μg)						
						12.4 ± 6.5 (placebo)						
Liutkus et al. [25]	5 countries (Multi-centre)	26 weeks	Randomised Controlled Study	165	Exenatide (55 ± 8)	Exenatide (6.3 ± 4.2)	Thiazolidinedione (given for 120 days before study), metformin + thiazolidinedione (given for at least 90 days before study). Participants continued their usual treatment for the duration of study.	Exenatide twice daily versus placebo	Exenatide showed superiority with respect to change in HbA1c (*p* < 0.001) and fasting serum glucose (*p* = 0.009) compared with placebo	There were no significant changes in fasting serum lipids between treatment groups.	Diastolic pressure was significantly reduced in both treatment groups, while systolic BP remained relatively unchanged from baseline values.	Mean reductions in body weight were not significantly different between treatments at endpoint
					Placebo (54 ± 9)	Placebo (6.4 ± 4.6)						
Apovian et al. [26]	USA (11 centres)	24 weeks	Randomised Controlled Study	194	54.8 ± 9.5	Exenatide (5.7 ± 5.5)	Metformin or sulfonylurea or both. Participants treated for at least 6 weeks before study and continued during study.	Exenatide twice daily + lifestyle modification programme placebo + lifestyle modification programme	Significantly more participants treated with exenatide + lifestyle modification had HbA1c ≤ 6.5% at end point compared with placebo + lifestyle modification (*p* = 0.001)	–	Exenatide + lifestyle modification was associated with significant decrease in systolic and diastolic BP at 24 weeks from baseline compared with placebo + lifestyle modification (*p* < 0.001; *p* = 0.04 )	Exenatide + lifestyle modification had significantly more weight loss compared with placebo + lifestyle modification (*p* < 0.01)
						Placebo (5.3 ± 5.1)						
Simo et al. [27]	14 countries (Multi-centre)	4½ years	Randomised Controlled Study	Exenatide (*n* = 511)	18–85	5.8 ± 4.8 (exenatide)	Metformin (participants treated before study and continued treatment during study)	Exenatide twice daily versus glimepiride	Symptomatic hypoglycaemia was reported during 1 year of study treatment by 13.5% (exenatide group) and 39.0% (glimepiride group) (*p* < 0.0001)	A significantly greater proportion of exenatide treated patients achieved the HDL-cholesterol goal than glimepiride treated patients at 36 months (*p* = 0.021)	Between-group differences were significantly in favor of exenatide for systolic BP (*p* < 0.001), diastolic BP (*p* = 0.023)	Between-group differences were significantly in favor of exenatide for body weight (*p* < 0.0001), waist circumference (*p* < 0.001).
				Glimepiride (n = 508)		5.5 ± 4.3 (glimepiride)						
Gallwitz et al. [28]	14 countries (128 centres)	4½ years	Randomised Controlled Study	Exenatide = 490	18–85	5.8 ± 4.8 (exenatide)	Metformin (participants treated before study and continued treatment during study)	Exenatide twice daily versus glimepiride	44% in exe natide group and 31% in glimepiride gp achieved HbA1c < 7% (*p* < 0.0001). 29% in exenatide gp and 18% in glimepiride group achieved HbA1c ≤ 6.5% (*p* = 0.0001). Significantly fewer patients reported hypoglycaemia in exenatide group compared with glimepiride group (*p* < 0.0001)	–	Systolic pressure decreased significantly in the exenatide group (*p* = 0.006) but not in the glimepiride group (*p* = 0.096). Heart rate increased in the exenatide group.	Significant decrease (*p* < 0.0001) in body weight in exenatide group compared with glimepiride group
				Glimepiride = 487		5.5 ± 4.3 (glimepiride)						
Gallwitz et al. [29]	Germany (Multi-centre)	26 weeks	Randomised Controlled Study	354	57 ± 10 (Exenatide)	5 ± 4 (Exenatide)	Metformin (participants treated before study and continued treatment during study)	Exenatide twice daily versus premixed insulin aspart (PIA)	HbA1c targets < 7% and <6.5% (exenatide noninferior to PIA). Hypoglcaemic epiosdes with blood glucose ≤ 3.0 mmols/L were less frequent with exenatide BID	–	–	Exenatide twice daily (weight loss = 4.1 ± 0.22 kg) PIA (weight gain = 1.0 ± 0.22 kg) SEM (*p* < 0.001)
					57 ± 9.9 (PIA)	5 ± 5 (PIA)						

HbA1c = Glycated haemoglobin; BP = Blood pressure; BMI = Body mass index; HDL = High density lipoprotein; LDL = Low density lipoprotein; PIA = Premixed insulin aspart; SEM = Standard error of mean.
